# Randomized Clinical Study of Laser-Assisted Delivery of Exosome Boosters for Postoperative Facial Scars and Facial Rejuvenation

**DOI:** 10.3390/life16020217

**Published:** 2026-01-28

**Authors:** Jei Youn Park, Jun Ho Park

**Affiliations:** Department of Plastic and Reconstructive Surgery, SMG-SNU Boramae Medical Center, Seoul National University College of Medicine, Seoul 07061, Republic of Korea

**Keywords:** exosome, skin booster, rejuvenation, scar, laser, anti-aging

## Abstract

Postoperative facial scars frequently remain aesthetically problematic despite advances in laser-based treatments, as residual inflammation and disorganized dermal remodeling often limit clinical outcomes. Exosome-based formulations have gained attention as biologically active adjuncts capable of influencing key wound-healing pathways, including inflammatory regulation, neovascularization, and extracellular matrix modulation. This randomized, controlled clinical study aimed to evaluate the short-term clinical effect of laser-assisted delivery of exosome skin boosters for postoperative facial scars and facial rejuvenation. Seventy-five patients with postoperative facial scars were randomly allocated to receive fractional non-ablative Nd:YAG laser treatment alone or in combination with either human-derived or plant-derived exosome skin boosters. All participants completed five treatment sessions at two-week intervals. Clinical outcomes were evaluated using validated scar assessment tools, including the modified Vancouver Scar Scale and the Patient and Observer Scar Assessment Scale, along with objective imaging analyses using Mark-Vu and ImageJ software. Compared with laser monotherapy, adjunctive exosome treatment was associated with numerically greater short-term improvements in scar appearance and reductions in grayscale intensity. Improvements in additional skin quality parameters, such as pigmentation uniformity, erythema, pore size, and fine wrinkles, were also observed in the exosome-treated groups. Clinical responses were comparable between human- and plant-derived exosome formulations, and no serious adverse events were reported. These findings indicate that exosome-based skin boosters may serve as a safe and well-tolerated biological complement to laser therapy for short-term improvement of postoperative facial scars and skin quality. Larger studies with longer follow-up are warranted to determine long-term efficacy and clinical durability.

## 1. Introduction

Advances in aesthetic medicine have increased patient expectations not only for scar improvement but also for overall enhancement of skin quality. Although a variety of treatment modalities—including fractional laser systems, injectable agents, and energy-based combination therapies—are widely used in clinical practice, their outcomes in postoperative scar management remain inconsistent [1,2,3]. Persistent inflammation, disorganized collagen remodeling, and residual pigmentation abnormalities frequently limit patient satisfaction following conventional interventions [4,5].

In parallel with scar management, skin rejuvenation has emerged as a major therapeutic goal in aesthetic practice. Traditional approaches such as chemical exfoliation, microneedling, and radiofrequency-based devices can stimulate dermal remodeling; however, their effects are often transient and highly variable [4]. To overcome these limitations, biologically active skin boosters have been introduced to modulate the wound-healing environment more directly. Injectable agents such as polynucleotides, calcium hydroxyapatite, poly-D,L-lactic acid, and acellular dermal matrices each promote extracellular matrix regeneration through distinct mechanisms, yet their clinical utility may be restricted by limited effects on pigmentation, risks of inflammatory reactions, or variability in integration within thin facial skin [6,7,8]. Collectively, these shortcomings highlight the need for biologic adjuncts capable of simultaneously regulating inflammation, vascular response, pigmentation, and extracellular matrix remodeling.

Exosomes, nanoscale extracellular vesicles released by both mammalian and plant cells, have gained increasing attention as mediators of tissue repair and regeneration [9]. By transporting bioactive cargos such as proteins, cytokines, and regulatory RNAs, exosomes participate in intercellular signaling pathways that influence inflammation, angiogenesis, and matrix organization [10]. Human-derived exosomes, particularly those originating from mesenchymal stem cells, have shown promising effects in wound healing and scar modulation; however, their broader clinical application is constrained by issues related to manufacturing cost, donor variability, and regulatory considerations [11,12].

Plant-derived exosomes have recently emerged as a potential alternative source of extracellular vesicles. These vesicles exhibit structural and functional similarities to mammalian exosomes while offering advantages in scalability, safety, and batch consistency [13]. Experimental studies suggest that plant-derived exosomes possess anti-inflammatory and antioxidant properties and may promote fibroblast activity and collagen synthesis [14]. Despite these encouraging findings, direct clinical comparisons between plant-derived and human-derived exosomes in scar management remain limited.

Fractional laser therapy may further enhance the biological activity of exosome-based treatments through laser-assisted drug delivery [15]. The creation of microthermal zones and transient epidermal channels facilitates deeper penetration of nanoscale vesicles into the dermis, enabling more efficient interaction with resident cells involved in wound repair [16]. Building on this rationale, the present study aimed to explore and compare the clinical effects of human-derived and plant-derived exosome skin boosters applied immediately after fractional non-ablative Nd:YAG laser treatment, with a focus on postoperative facial scars and selected facial rejuvenation parameters. Accordingly, this study was designed as an exploratory randomized clinical investigation to assess short-term clinical and optical changes rather than definitive long-term scar remodeling.

## 2. Materials and Methods

### 2.1. Study Design and Participants

This prospective, randomized, controlled clinical study was designed as an exploratory investigation to evaluate short-term clinical outcomes. The study was conducted at Seoul National University Boramae Medical Center between September and December 2025 after approval by the Institutional Review Board (IRB No. 20202524). Written informed consent was obtained from all participants, and the study adhered to the Declaration of Helsinki.

Seventy-five patients (aged 20–75 years) with clinically visible postoperative facial scars aged 3–6 weeks were enrolled. Scars were enrolled during the early remodeling phase, a period characterized by ongoing inflammation and active extracellular matrix reorganization. The rationale for this timing was to evaluate whether adjunctive biological interventions could modulate early scar appearance rather than to assess outcomes in fully matured scars. All scars were linear postoperative facial scars resulting from elective facial surgical procedures, including benign lesion excision and reconstructive closure after minor oncologic surgery. Hypertrophic or keloid scars were excluded. Exclusion criteria included hypertrophic or keloid scarring tendencies, active dermatologic infection, systemic immunosuppression, pregnancy, or recent scar-modifying treatments. Participants were randomly allocated in equal numbers (*n* = 25 per group) to one of three treatment arms: fractional non-ablative Nd:YAG laser alone (Group I), laser plus human-derived exosome booster (Group II), or laser plus plant-derived exosome booster (Group III). Randomization was performed using a computer-generated random sequence with a 1:1:1 allocation ratio. Allocation concealment was maintained by having the randomization list managed by a dedicated research nurse who was not involved in treatment administration or outcome assessment. Group assignment was disclosed to the treating clinician only at the time of intervention. Outcome assessors (three plastic surgeons) and image analysts were blinded to group allocation.

### 2.2. Treatment Protocol

All participants underwent five treatment sessions at two-week intervals. Fractional non-ablative laser treatment was performed using a 1064-nm Nd:YAG device in microlens array mode with standardized parameters. To facilitate laser-assisted drug delivery, an additional toning mode was applied across the facial surface to generate multiple transient microchannels. Immediately after each session, participants in Groups II and III received topical application of the assigned exosome booster, which was gently massaged to enhance dermal penetration.

### 2.3. Exosome Preparation

The plant-derived exosome formulation (Exome^®^, Cosmedician Co., Ltd., Busan, Republic of Korea) was obtained from aseptically cultured plant tissue under controlled conditions, followed by purification of extracellular vesicles using established filtration and ultracentrifugation methods. The human-derived exosome formulation (Exomide^®^, Cosmedician Co., Ltd., Busan, Republic of Korea) was prepared from conditioned media of adipose-derived mesenchymal stem cells and contained nanoscale vesicles enriched with regenerative growth factors and cytokines. Both formulations underwent quality control procedures to confirm vesicle size distribution and structural integrity. It should be noted that both formulations are commercial products containing extracellular vesicles along with additional bioactive components, and the present study was not designed to isolate the effects of exosomes from other formulation constituents.

### 2.4. Outcome Assessment

Clinical evaluations were conducted at baseline and two weeks after completion of the final treatment session. Subjective scar assessment was performed using the modified Vancouver Scar Scale and the Patient and Observer Scar Assessment Scale [17]. Observer-based evaluations were independently completed by three blinded plastic surgeons, while patient-reported outcomes were obtained using the patient component of the POSAS. Mean values derived from observer and patient assessments were used for subsequent analyses.

Objective evaluation of scar morphology and skin quality was performed using a non-contact three-dimensional optical imaging system (Mark-Vu^®^, PSI PLUS, Suwon, Republic of Korea). This system was used to quantify grayscale intensity as an objective optical indicator of scar surface characteristics, along with additional parameters including pigmentation, pore size, erythema, and wrinkle depth. Grayscale intensity values were interpreted as reflecting surface reflectance and optical uniformity of the scar area, with lower values indicating smoother texture and reduced visual conspicuity. To further characterize surface heterogeneity, complementary grayscale-based pixel intensity analysis was conducted using ImageJ version 1.54 software (NIH, Bethesda, MD, USA). Standardized clinical photographs were acquired under identical imaging conditions, and regions of interest were consistently defined over scarred areas using anatomical landmarks. Mean grayscale values within each region were analyzed on an 8-bit scale, and changes in pixel intensity distribution were compared between baseline and post-treatment images.

### 2.5. Statistical Analysis

Statistical analyses were performed using SPSS version 25.0 (IBM Corp., Armonk, NY, USA). Continuous data are presented as mean ± standard deviation. Within-group comparisons were analyzed using paired *t*-tests, and between-group differences were assessed using one-way analysis of variance with Tukey’s post hoc test. A two-sided *p*-value < 0.05 was considered statistically significant. No adjustment for multiple comparisons was applied, and all *p*-values should therefore be interpreted descriptively. In addition, the study was not powered to detect between-group differences, and statistical significance should not be interpreted as evidence of clinical superiority or definitive efficacy. Given the exploratory nature of the study and the number of outcome measures assessed, statistical analyses were intended to generate hypothesis-supporting evidence rather than definitive comparative conclusions. The primary endpoint was predefined, for exploratory purposes, as the change in grayscale intensity (GSI) measured by Mark-Vu from baseline to two weeks after the final session. Secondary endpoints included changes in mVSS, POSAS (patient and observer), and additional Mark-Vu skin quality parameters (pigmentation, pores, redness, and wrinkle indices).

## 3. Results

**Figure 1:**
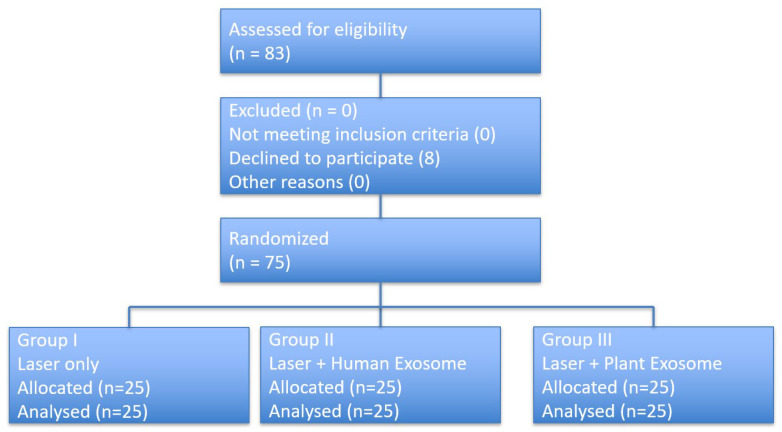
CONSORT flow diagram of participant enrollment, randomization, intervention allocation, follow-up, and analysis.

A total of 75 patients were randomized in a 1:1:1 ratio and allocated to the laser-only group, laser plus human-derived exosome group, or laser plus plant-derived exosome group. Participant flow is summarized in Figure 1. All randomized participants received the allocated intervention, completed the treatment protocol, and were included in the final analysis.

### 3.1. Scar-Related Outcomes and Objective Imaging Analysis

**Figure 2:**
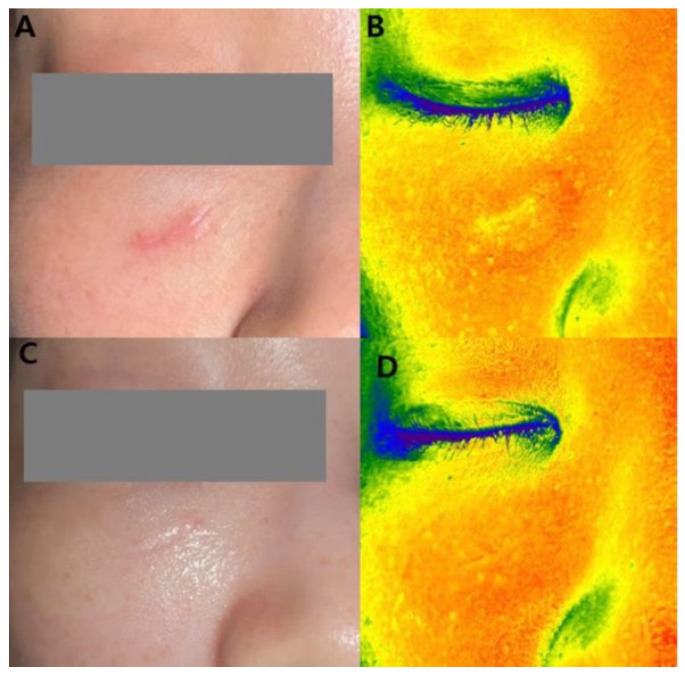
A 32-year-old female patient underwent laser treatments combined with five sessions of plant-derived exosome treatments. (**A**) Baseline clinical photograph. (**B**) Baseline Mark-Vu grayscale intensity analysis showing increased signal intensity corresponding to the scar area. (**C**) Clinical photograph after five treatment sessions. (**D**) Post-treatment Mark-Vu Grayscale intensity analysis showing reduction in grayscale intensity.

Objective imaging using the Mark-Vu analysis system demonstrated changes in grayscale intensity across all treatment groups (Table 1, Figure 2). Mean grayscale intensity decreased from 165.8 ± 12.4 to 162.9 ± 11.8 in the laser-only group, from 171.6 ± 13.1 to 160.3 ± 12.5 in the laser plus human-derived exosome group, and from 168.9 ± 12.7 to 155.8 ± 11.9 in the laser plus plant-derived exosome group. Between-group comparisons showed statistically significant differences in grayscale intensity change among the three groups (*p* = 0.01).

Clinical scar assessments using validated scales are summarized in Table 1. Mean modified Vancouver Scar Scale (mVSS) scores decreased from 5.1 to 4.4 in the laser-only group, from 5.3 to 2.8 in the human-derived exosome group, and from 4.9 to 3.1 in the plant-derived exosome group. Intergroup comparison of mVSS score changes demonstrated a statistically significant difference (*p* = 0.03).

Observer-reported POSAS scores decreased from 20.8 to 17.7 in the laser-only group, from 20.6 to 15.2 in the human-derived exosome group, and from 21.1 to 15.8 in the plant-derived exosome group, with a statistically significant difference among groups (*p* = 0.03). Patient-reported POSAS scores decreased from 13.6 to 11.1, from 14.5 to 11.7, and from 13.1 to 10.4 in Groups I, II, and III, respectively; however, intergroup differences in patient-reported score changes were not statistically significant (*p* = 0.26).

### 3.2. Rejuvenation-Related Skin Quality Outcomes

**Figure 3:**
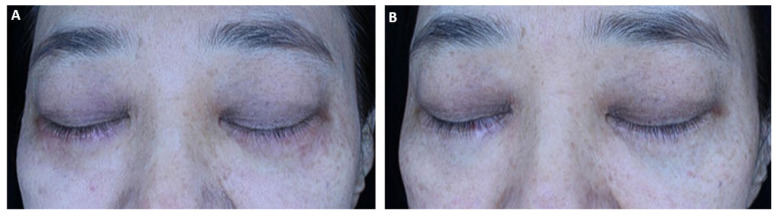
A 46-year-old female patient underwent laser treatments combined with five sessions of human-derived exosome treatments. (**A**) Baseline clinical photograph showing periocular hyperpigmentation and uneven skin tone prior to treatment. (**B**) Post-treatment photograph demonstrating visible improvement in pigmentation and overall skin uniformity.

Changes in skin quality parameters assessed using the Mark-Vu imaging system are presented in Table 2 and Figure 3. Mean pigmentation (melanin) values decreased by 0.4 ± 0.6 in the laser-only group, 1.1 ± 0.8 in the human-derived exosome group, and 1.0 ± 0.7 in the plant-derived exosome group, with statistically significant intergroup differences (*p* = 0.03).

Mean pore size indices decreased by 0.5 ± 0.6, 1.2 ± 0.9, and 1.1 ± 0.8 in Groups I, II, and III, respectively, with a significant difference among groups (*p* = 0.04). Redness scores increased by 0.2 ± 0.5 in the laser-only group and decreased by 2.6 ± 1.1 and 2.8 ± 1.2 in the human-derived and plant-derived exosome groups, respectively, with statistically significant intergroup differences (*p* = 0.01). Wrinkle depth indices decreased by 0.4 ± 0.5, 0.9 ± 0.6, and 0.8 ± 0.6 in Groups I, II, and III, respectively, with a significant difference among groups (*p* = 0.02).

Within-group pre- to post-treatment changes in outcome measures are summarized in Table 1 and Table 2. No statistically significant between-group differences were observed between Group II and Group III across assessed outcomes (all *p* > 0.05).

### 3.3. Safety Outcomes

No serious adverse events were reported during the study period. Mild, transient erythema and a brief burning sensation were reported by two participants in the human-derived exosome group and resolved spontaneously without medical intervention. No adverse reactions were observed in the plant-derived exosome group. All participants completed the treatment protocol without discontinuation.

## 4. Discussion

Postoperative scar management remains a persistent challenge in aesthetic and reconstructive practice [18]. Although modalities such as fractional laser resurfacing, chemical peeling, and radiofrequency-based treatments can improve scar appearance to some extent, their clinical outcomes are often limited by sustained inflammation, irregular collagen remodeling, and post-inflammatory pigmentary changes [19,20]. Non-ablative fractional lasers induce controlled dermal injury to stimulate repair; however, treatment responses are variable and frequently modest across different patient populations [21,22].

In this context, biologically active adjuncts that can modulate the wound-healing microenvironment have gained increasing attention [23]. Exosomes, nanosized extracellular vesicles secreted by mammalian and plant cells, play an important role in intercellular communication and tissue repair [24]. Human mesenchymal stem cell-derived exosomes contain bioactive cargos, including growth factors, cytokines, and regulatory RNAs, which influence fibroblast activity, angiogenesis, and extracellular matrix remodeling [25]. Experimental studies have shown that exosomal microRNAs such as miR-21, miR-23a, and miR-125b suppress profibrotic signaling pathways, including TGF-β/Smad signaling, thereby limiting myofibroblast differentiation and excessive collagen deposition [26]. Despite these promising biological effects, the clinical translation of human-derived exosomes is constrained by manufacturing costs, donor variability, and regulatory considerations [27].

The present randomized study demonstrated that adjunctive application of both human-derived and plant-derived exosome boosters was associated with greater improvements in scar-related outcomes compared with laser monotherapy, as reflected by reductions in mVSS and POSAS scores and by objective imaging parameters. Importantly, clinical responses were comparable between the two exosome formulations.

To avoid misleading interpretation, the present findings should not be viewed as evidence of definite efficacy or superiority. Rather than establishing clinical effectiveness, this study aimed to explore short-term trends and feasibility of adjunctive exosome application in the early postoperative remodeling phase. Accordingly, while numerical improvements were observed, these results should be interpreted within the context of an exploratory, hypothesis-generating clinical investigation rather than as confirmatory evidence supporting therapeutic recommendations.

Plant-derived exosomes offer several practical advantages, including scalability, low immunogenicity, and consistent production under controlled conditions [12,28]. Preclinical investigations have reported antioxidant and anti-inflammatory properties of plant-derived vesicles, along with their ability to promote fibroblast proliferation and collagen synthesis [28]. In the present study, the use of aseptically cultured plant tissue provided a standardized source of extracellular vesicles, supporting the feasibility of plant-derived exosomes for clinical application.

Beyond scar remodeling, improvements were also observed in multiple skin quality parameters, including pigmentation, erythema, pore size, and wrinkle depth. These findings suggest that exosome-based boosters may exert broader effects on skin homeostasis. Mechanistically, exosomes have been reported to influence melanogenic signaling, oxidative stress responses, and vascular reactivity, while simultaneously enhancing dermal extracellular matrix organization [9,29,30]. Such multi-axis biological activity may explain the observed improvements in both scar-related and rejuvenation-related outcomes.

The synergistic use of fractional non-ablative laser treatment and exosome application may further contribute to these effects through laser-assisted drug delivery [22,31]. Laser-induced microthermal zones and transient epidermal channels enhance dermal permeability, facilitating the penetration and cellular uptake of nanoscale vesicles [5,32]. This laser-mediated microenvironment may potentiate exosome bioactivity by promoting interactions with dermal fibroblasts, endothelial cells, and immune cells involved in wound repair.

Several additional limitations of this study should be acknowledged. First, objective morphometric measurements of scar length and width were not systematically recorded at baseline. Although linear dimensions may provide structural information, postoperative facial scars are commonly assessed in clinical practice using validated multidimensional instruments such as the modified Vancouver Scar Scale and the Patient and Observer Scar Assessment Scale, which integrate vascularity, pigmentation, pliability, thickness, and patient-reported symptoms. Accordingly, the primary outcome measures of this study focused on established clinical scar assessment scales and complementary optical imaging parameters rather than isolated geometric dimensions. Given that the study targeted scars during the early postoperative remodeling phase, the absence of baseline morphometric measurements may have influenced short-term appearance-related assessments; however, this limitation is less likely to affect long-term structural remodeling outcomes. Future studies should incorporate standardized morphometric measurements alongside validated scar scales to enable more comprehensive baseline characterization and quantitative adjustment.

In addition, no formal sample size or power calculation was performed prior to study initiation, and the sample size was determined pragmatically based on feasibility considerations. As this study was designed as an exploratory, hypothesis-generating trial rather than a confirmatory efficacy study, formal power assumptions were not applied. Consequently, the study may be underpowered to detect small between-group differences, particularly between the two exosome-treated groups, and the findings should be interpreted as preliminary. Finally, this exploratory trial was not prospectively registered in a public clinical trial registry; therefore, the results should be interpreted with caution, and future confirmatory trials should be prospectively registered with clearly prespecified endpoints.

Future investigations should include longer follow-up periods of at least six months to assess sustained remodeling, scar recurrence, and delayed pigmentary changes. Incorporation of histological evaluation would allow direct correlation between clinical findings and dermal structural alterations. Larger, adequately powered trials involving more diverse ethnic populations are warranted to confirm these preliminary observations. Comparative studies with other regenerative injectables and mechanistic investigations into exosome-mediated regulation of melanogenesis, vascular normalization, and extracellular matrix dynamics may further elucidate the clinical role of exosome-based skin boosters.

In summary, adjunctive use of both human-derived and plant-derived exosome boosters in combination with fractional laser therapy was associated with improved postoperative scar outcomes and enhanced skin quality in this randomized clinical study. The comparable performance and favorable safety profile of plant-derived exosomes support their further evaluation as a scalable biological adjunct in aesthetic and regenerative dermatology.

## 5. Conclusions

In this randomized clinical study, adjunctive use of human-derived and plant-derived exosome boosters in combination with fractional laser therapy was associated with short-term improvements in postoperative facial scar appearance and selected skin quality parameters. The comparable performance of plant-derived exosomes suggests their potential feasibility as a scalable biological adjunct. However, these findings should be interpreted as preliminary, and larger studies with longer follow-up are required to determine long-term efficacy and clinical durability.

## Figures and Tables

**Table 1:** Pre- and post-treatment scar outcomes in each group with intergroup comparison (Scar).

	Group I (Laser Only)	Group II (Laser + Human Exosome)	Group III (Laser + Plant Exosome)	*p*-Value †
**Mean mVSS**				
**Pre**	5.1	5.3	4.9	
**Post**	4.4	2.8	3.1	
**Improvement**	0.7	2.5	2.8	0.03 *
**Mean POSAS (Patients)**				
**Pre**	13.6	14.5	13.1	
**Post**	11.1	11.7	10.4	
**Improvement**	2.5	2.8	2.7	0.26
**Mean POSAS (Observer)**				
**Pre**	20.8	20.6	21.1	
**Post**	17.7	15.2	15.8	
**Improvement**	3.1	5.4	5.3	0.03 *
**Grayscale intensity (GSI)**				
**Pre**	165.8	171.6	168.9	
**Post**	162.9	160.3	155.8	
**Improvement**	2.9	11.3	13.1	0.01 *

mVSS: modified Vancouver Scar Scale; POSAS: Patient and Observer Scar Assessment Scale; * Statistically significant difference among groups (*p* < 0.05); † *p*-values calculated using one-way ANOVA for between group comparisons.

**Table 2:** Pre- and post-treatment scar outcomes in each group with intergroup comparison (rejuvenation).

	Group I (Laser Only)	Group II (Laser + Human Exosome)	Group III (Laser + Plant Exosome)	*p*-Value †
**Pigmentation (Melanin)**				
**Pre**	51.3	48.3	49.9	
**Post**	50.9	47.2	48.9	
**Improvement**	0.4	1.1	1.0	0.03 *
**Pores**				
**Pre**	47.1	50.1	48.5	
**Post**	46.6	48.9	47.4	
**Improvement**	0.5	1.2	1.1	0.04 *
**Redness**				
**Pre**	14.9	13.0	13.6	
**Post**	15.1	10.4	10.8	
**Improvement**	−0.2	2.6	2.8	0.01 *
**Wrinkle**				
**Pre**	16.7	16.4	16.7	
**Post**	16.3	15.5	15.9	
**Improvement**	0.4	0.9	0.8	0.02 *

* Statistically significant difference among groups (*p* < 0.05); † *p*-values calculated using one-way ANOVA for between group comparisons.

## Data Availability

The data will be made available on request.

## Abbreviations

The following abbreviations are used in this manuscript:

mVSS	Modified Vancouver Scar Scale
POSAS	Patient and Observer Scar Assessment Scale
GSI	Grayscale Intensity
Nd:YAG	Neodymium-Doped Yttrium Aluminum Garnet
MLA	Microlens Array
ROI	Region of Interest
EV	Extracellular Vesicle
MSC	Mesenchymal Stem Cell
IL	Interleukin
TGF-β	Transforming Growth Factor-beta
VEGF	Vascular Endothelial Growth Factor
SD	Standard Deviation
ANOVA	Analysis of Variance
IRB	Institutional Review Board
SPSS	Statistical Package of the Social Science

## References

[1] Elesawy F., Habashy A., Hamed N. (2023). Acne Scars and Fractional Laser: A Comprehensive Review. Benha J. Appl. Sci..

[2] Yue S., Ju M., Su Z. (2022). A Systematic Review and Meta-Analysis of Botulinum Toxin A in the Prevention of Postoperative Facial Scars. Aesthetic Plast. Surg..

[3] Almukhadeb E., Binkhonain F., Alkahtani A., Alhunaif S., Altukhaim F., Alekrish K. (2023). Dermal Fillers in the Treatment of Acne Scars: A Review. Ann. Dermatol..

[4] Zhou C.J., Guo Y. (2024). Collagens in Normal Skin and Pathological Scars: Current Understanding and Future Perspectives. Front. Med..

[5] Chen S.X., Cheng J., Watchmaker J., Dover J.S., Chung H.J. (2022). Lasers and Energy-Based Devices for Skin Rejuvenation and Scar Treatment: A Histologic Review. Dermatol. Surg..

[6] Van Loghem J. (2025). Calcium Hydroxylapatite in Regenerative Aesthetics: Mechanistic Insights and Mode of Action. Aesthetic Surg. J..

[7] Lee K.W.A., Chan L.K.W., Lee A.W.K., Lee C.H., Wong S.T.H., Yi K.-H. (2024). Poly (D,L-Lactic Acid) Applications in Dermatology: A Literature Review. Polymers.

[8] Tognetti L., Pianigiani E., Ierardi F., Lorenzini G., Casella D., Liso F.G., De Pascalis A., Cinotti E., Rubegni P. (2021). Human Acellular Dermal Matrices in Advanced Wound Healing and Surgery. Dermatol. Ther..

[9] Dal’Forno-Dini T., Birck M.S., Rocha M., Bagatin E. (2025). Exploring the Reality of Exosomes in Dermatology. An. Bras. Dermatol..

[10] Hu J.-C., Zheng C.-X., Sui B.-D., Liu W.-J., Jin Y. (2022). Mesenchymal Stem Cell-Derived Exosomes in Cutaneous Wound Healing. World J. Stem Cells.

[11] Hajialiasgary Najafabadi A., Soheilifar M.H., Masoudi-Khoram N. (2024). Exosomes in Skin Photoaging: Biological Functions and Therapeutic Opportunities. Cell Commun. Signal..

[12] Bai C., Zhang X., Li Y., Qin Q., Song H., Yuan C., Huang Z. (2024). Research Progress and Challenges of Plant-Derived Exosome-Like Nanoparticles. Biomed. Pharmacother..

[13] Cho J.H., Hong Y.D., Kim D., Park S.J., Kim J.S., Kim H.-M., Yoon E.J., Cho J.-S. (2022). Plant-Derived Exosomes as Bioactive Substances for Skin Application. Appl. Biol. Chem..

[14] Commander S.J., Chamata E., Cox J., Dickey R.M., Lee E.I. (2016). Update on Postsurgical Scar Management. Semin. Plast. Surg..

[15] Chi P.-L., Lee G.-S., Huang P.P.-H. (2024). Laser-Assisted Drug Delivery. Updates on Lasers in Dermatology.

[16] Haghsay Khashechi E., Afaghmehr A., Heidarizade N., Barfar A., Shokri J. (2024). Laser-Mediated Transdermal Drug Delivery. AAPS PharmSciTech.

[17] Park J.W., Koh Y.G., Shin S.H., Choi Y.-J., Kim W.-S., Yoo H.H., Lee J.O., Jang Y.N., Kim J., Li K. (2022). Review of Scar Assessment Scales. Med. Lasers..

[18] Quan T.S., Anh L.V., Hung T.Q., Vuong N.L. (2023). The Efficacy of Intense Pulsed Light for Keloids and Hypertrophic Scars. J. Lasers Med. Sci..

[19] Agrawal K., Belgaumkar V.A., Chavan R.B., Pradhan S.N. (2024). Evaluating the Pros and Cons of Fractional CO_2_ Laser versus Microneedling in Atrophic Acne Scars in Skin of Color: A Split-Face Study. Indian Dermatol. Online J..

[20] An J.K., Kim Y.H. (2024). Clinical Application of Silicone Sheets and Gel in Postoperative Scar Management. J. Wound Manag. Res..

[21] Wang Y., Zheng Y., Cai S. (2022). Non-Ablative Fractional Laser versus Nd:YAG Laser for Enlarged Facial Pores. Lasers Med. Sci..

[22] Taudorf E.H., Danielsen P.L., Paulsen I.F., Togsverd-Bo K., Dierickx C., Paasch U., Haedersdal M. (2015). Non-Ablative Fractional Laser for Mature Burn Scars. Lasers Surg. Med..

[23] Zhao H., Li Z., Wang Y., Zhou K., Li H., Bi S., Wang Y., Wu W., Huang Y., Peng B. (2023). Bioengineered MSC-Derived Exosomes in Skin Repair. Front. Cell Dev. Biol..

[24] Zhang Y., Yan W., Wu L., Yu Z., Quan Y., Xie X. (2025). Exosome-Loaded Hydrogels for Tissue Repair Applications. Front. Bioeng. Biotechnol..

[25] Lou G., Chen Z., Zheng M., Liu Y. (2017). MSC-Derived Exosomes as Therapeutic Strategy. Exp. Mol. Med..

[26] Lu Y., Liu Z., Zhang Y., Wu X., Bian W., Shan S., Yang D., Ren T. (2023). METTL3-Mediated Fibrosis via miR-21/PTEN Pathway. Respir. Res..

[27] Pamulang Y.V., Oontawee S., Rodprasert W., Padeta I., Sa-Ard-lam N., Mahanonda R., Osathanon T., Somparn P., Pisitkun T., Torsahakul C. (2025). Upscaling and Bioactivity Screening of Canine MSC Exosomes. Sci. Rep..

[28] Wu W., Zhang B., Wang W., Bu Q., Li Y., Zhang P., Zeng L. (2024). Plant-Derived Exosome-Like Nanovesicles in Chronic Wound Healing. Int. J. Nanomed..

[29] Karabay A.Z., Barar J., Hekmatshoar Y., Rahbar Saadat Y. (2025). Therapeutic Potential of Plant-Derived Exosomes. Biomolecules.

[30] Arabpour M., Saghazadeh A., Rezaei N. (2021). Anti-Inflammatory Effects of MSC-Derived Exosomes. Int. Immunopharmacol..

[31] Begum J., Riyaz M., Hyma P. (2024). Laser-Assisted Drug Delivery: A Comprehensive Review. J. Adv. Sci. Res..

[32] Huth S., Huth L., Marquardt Y., Cheremkhina M., Heise R., Baron J.M. (2022). MMP-3 in Fractional Laser-Promoted Wound Healing. Lasers Med. Sci..

